# Exploring the therapeutic potential of forkhead box O for outfoxing COVID-19

**DOI:** 10.1098/rsob.210069

**Published:** 2021-06-09

**Authors:** Pradeep Singh Cheema, Deeptashree Nandi, Alo Nag

**Affiliations:** Department of Biochemistry, University of Delhi, South Campus, Biotech Building, 2nd Floor, Benito Juarez Road, Dhaula Kuan, New Delhi 110021, India

**Keywords:** COVID-19, coronavirus, FoxO, inflammation, cytokines, immune response

## Abstract

The COVID-19 pandemic has wreaked unprecedented societal havoc worldwide. The infected individuals may present mild to severe symptoms, with nearly 20% of the confirmed patients impaired with significant complications, including multi-organ failure. Acute respiratory distress imposed by SARS-CoV-2 largely results from an aggravated cytokine storm and deregulated immune response. The forkhead box O (FoxO) transcription factors are reported to play a significant role in maintaining normal cell physiology by regulating survival, apoptosis, oxidative stress, development and maturation of T and B lymphocytes, secretion of inflammatory cytokines, etc. We propose a potent anti-inflammatory approach based on activation of the FoxO as an attractive strategy against the novel coronavirus. This regime will be focused on restoring redox and inflammatory homeostasis along with repair of the damaged tissue, activation of lymphocyte effector and memory cells. Repurposing FoxO activators as a means to alleviate the inflammatory burst following SARS-CoV-2 infection can prove immensely valuable in the ongoing pandemic and provide a reliable groundwork for enriching our repertoire of antiviral modalities for any such complication in the future. Altogether, our review highlights the possible efficacy of FoxO activation as a novel arsenal for clinical management of COVID-19.

## Graphical abstract

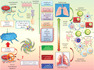

## 1. Introduction

The severe acute respiratory syndrome coronavirus 2 (SARS-CoV-2) has wreaked havoc across the globe since its emergence in late 2019 [1]. The spread of the coronavirus disease pushed the World Health Organization (WHO) to declare COVID-19 a worldwide pandemic. Proved to be exceptionally contagious with a current basic reproduction number (*R*_0_) of approximately 3, this global catastrophe demands immediate and adequate attention [2]. Estimates clearly state that at least 60–80% of a given population is falling prey to the grasp of this pandemic, with high fatalities. We have already witnessed a rapid downward spiralling of health sectors across all nations with multiple countries failing to accommodate the soaring number of cases. However, the far-reaching impacts of this calamity on other aspects of civilization, such as economy, are beyond our comprehension. The clinical manifestations of COVID-19 appear mild in most of those infected; however, nearly 20% of the patients suffer from more severe symptoms, including acute respiratory distress syndrome, septic shock and systemic failure. This often culminates in the death of the patient. Although physicians have been trying out various modes of treatment, clinical management of this disease is primarily symptomatic.

The Forkhead Box O (FoxO) subfamily of transcription factors has been elucidated to play critical functions in pulmonary homeostasis apart from their involvement in various cellular biochemical functions [3]. Their importance in maintaining normal lung physiology is highlighted by the appearance of lung abnormalities upon loss of FoxOs. In concordance, restoration of their activity led to the attenuation of such pathological states [4]. In fact, in-depth analysis of conditions such as idiopathic pulmonary fibrosis (IPF) has shown FoxO to be rendered inactive in the diseased tissue relative to the normal counterpart [4,5]. FoxO is also known to play an impressive role in regulating the immune and inflammatory response of the human body against various infections [6,7]. Normally, FoxOs upregulate various pro-inflammatory cytokines such as interleukin (IL)-1β and IL-9, Toll-like receptor (TLR)1 and TLR4, etc., which not only modulate the host inflammatory reaction but also alter the innate immune response [8,9]. Moreover, FoxO factors are also essential for the adaptive immune functions including maturation and differentiation of B and T lymphocytes [10,11]. Thus, the interplay of FoxOs with SARS-CoV-2 presents a possible vital coalition, which can be targeted to tackle the detrimental inflammatory upsurge post-SARS-CoV-2 infection. This review thus underscores the importance of FoxO proteins in mechanistically regulating the host inflammatory and immunological response to SARS-CoV-2. We also suggest a potential outlook into the promising role of these transcription factors in restraining the pathogenesis of novel coronavirus.

## 2. SARS-CoV-2 exacerbates host inflammation

The coronavirus (CoV) is a member of the Coronavirinae subfamily and features a typical crown-like appearance. It has four distinct structural proteins that play a crucial role in viral propagation and pathogenesis. The membrane (M) protein has three transmembrane domains, which are involved in shaping the virion, inducing membrane curvature and binding to the nucleocapsid whereas the envelope (E) protein is important for virion assembly and subsequent release. The nucleocapsid (N) protein aids in packaging of the viral genome into the nucleocapsid while the spike (S) protein forms trimeric spikes that facilitate attachment and fusion of the virus to the host receptor [12]. SARS-CoV-2 has been shown to invade host cells through recognition and binding to the angiotensin-converting enzyme 2 (ACE2) by its S-protein [13]. ACE2 is majorly expressed in the lung and heart tissues. It hydrolyses Angiotensin (Ang)-I and Ang-II to Ang-(1–7), which binds to the Mas receptor and imparts vascular protective, vasodilating, anti-fibrotic, anti-proliferative and anti-inflammatory effects [14]. Unsurprisingly, elevated level of Ang-II promotes vascular permeability and oedema in the lungs, whereas utilization of a selective Ang-II receptor antagonist losartan demonstrated alleviation of pulmonary injury and associated inflammation. Therefore, the suppression of ACE2 expression by SARS-CoV-2 S-protein promotes austere lung failure and blockage of the renin–angiotensin–aldosterone pathway attenuated such phenotype [15]. A majority of the COVID-19 patients have been found to exhibit lymphocytopaenia, which was further connected to a decreased CD4^+^/CD8^+^ cell population. Analysis of a panel of serum cytokine levels including interferon (IFN)-α, IFN-γ, IL-1β, IL-2, IL-4, IL-5, IL-6, IL-8, IL-10, IL-12 and IL-17 in these patients revealed a significant elevation in the levels of IL-6 and IL-8 only, which were linked to the severity of disease [16,17]. The role of lung macrophages in initiating the cytokine inflow in the primary stages of the disease has also been suggested, thus indicating a cross-talk among various cell signalling molecules to manifest the severity of SARS-CoV-2 infection [18,19]. In fact, CD4^+^/CD8^+^ lymphocytes were also observed to be majorly dwindled in SARS-CoV pathogenesis [20]. SARS-CoV-2 infection triggers pro-inflammatory cytokines to activate the inflammatory cells. Immunity against the virus is usually developed through production of IFN-γ and IL-17 by CD4^+^ cells [21]. Following pathogenesis of SARS-CoV-2, the resultant cytokine storm, which is basically a heightened release of cytokines, is the primary cause underlying T-cell depletion, pulmonary inflammation, lung dysfunction and multiple organ failure in the host [22]. The detrimental snowball outcome of this is granulocytosis, which is responsible for a strong burst of reactive oxygen species (ROS) by the immune cells. This stimulates oxidative stress, which complements prominent features associated with macrophage activation syndrome [23]. Such patients usually display elevated levels of pro-inflammatory cytokines including interleukins, granulocyte colony-stimulating factor (gCSF), IFN-γ inducible protein, tumour necrosis factor (TNF)-α, monocyte chemoattractant protein and macrophage inflammatory protein. In individuals with COVID-19, the enhanced serum level of the pleiotropic cytokine IFN-γ transcriptionally stimulates the production of IL-6 from the monocytes [24]. In addition to this direct instigation, IFN-γ also functions to activate a downstream repertoire of inflammatory cascade via the Janus kinase (JAK) pathway [25]. IFN-γ is known to bind and phosphorylate the c-Jun N-terminal kinase (JNK) 1 and 2 proteins, which then heterodimerize and further phosphorylate and activate the signal transducer and activator of transcription (STAT) pathway. The activated STAT proteins in turn transcriptionally upregulate various genes known as interferon-stimulated genes (ISGs) involved in multiple inflammatory processes [26].

A thorough understanding of the most relevant context for introducing an anti-inflammatory therapy in juxtaposition to some sort of antiviral module may provide excellent opportunities to manage the symptoms of COVID-19. Such a regime must be able to curb the hyper-inflammation without altering the host's efficiency in mounting an immune response for virus clearance. In this aspect, it will be interesting to see whether exploiting FoxO can successfully impart protection against the SARS-CoV-2 virus-associated inflammatory response in addition to restoring redox equilibrium and favouring tissue repair.

## 3. Overview of FoxO factors: a master regulator

The members of the FoxO class belong to a large superfamily of forkhead transcription factors that are characterized by their conserved DNA binding ‘forkhead box’ domain. The FoxO proteins are expressed in nearly all tissues. They act as regulators of pleiotropic functions within the cells that have considerable consequences in host health and disease [3]. FoxOs transcriptionally modulate expression of a multitude of downstream effector genes that are involved in cellular proliferation, cell cycle arrest, apoptosis, genomic repair, metabolic balance, redox homeostasis and resistance to oxidative stress [27]. The activation of these *bona fide* tumour suppressors depends on the growth factor signalling cascade. Their prime upstream regulator is the phosphoinositide-3-kinase/protein kinase B (PI3K/Akt) pathway [28]. Sequential phosphorylation of FoxOs in the presence of growth factors results in their cytoplasmic sequestration or ubiquitination, thus rendering them inactive. In the absence of growth factors, phosphatase and tensin homologue (PTEN) abolishes the PI3K/Akt-mediated phosphorylation of FoxO, thus leading to its dephosphorylation and subsequent nuclear shuttling. Once in the nucleus, FoxO factors are involved in the transcriptional regulation of several downstream target genes [29,30].

The deregulation of FoxO activity is frequented in diverse pathological conditions, including diabetes, infertility, neurodegenerative disorders, carcinomas and immune system malfunctions. Recent studies have disclosed that FoxOs may also impact longevity and delay the process of ageing in humans [31]. Remarkably, a few of these diseased states (such as diabetes) and older age are ascertained risk factors involved in the respiratory distress mediated by SARS-CoV-2 [32]. Notably, FoxO factors are intimately associated with development and maintenance of normal lung tissue architecture [33]. The therapeutic outcome of FoxO activation has been reported in animal models with lung ailments, including IPF. FoxO downregulation was distinctly observed in IPF fibroblasts. Furthermore, FoxO knockdown in healthy mice augmented fibrosis while obliterating pulmonary function. Pharmacological restoration of FoxO3 activity, in part, successfully reverted the IPF phenotype [4,5], thereby reinforcing FoxO as a potent therapeutic target for pulmonary catastrophes. In agreement, pharmacological reconstitution of FoxO1 in models of pulmonary hypertension directly promotes reversal of the adverse lung vasculature and related clinical features [34]. FoxO3 modulations have also been linked to different pathological features of pulmonary fibrosis. Analogous to this notion, the amount of inactive FoxO3 is substantially higher in IPF fibroblasts compared to healthy lung fibroblasts. Additionally, FoxO3 repressed macrophages from secreting IL-13. This helped to slow down pulmonary inflammation and fibrosis. Together, such evidence indicated that FoxOs are potential targets for inhibiting lung fibrosis and aiding in tissue repair of the damaged lung, which is usually encountered in critically ill COVID-19 individuals [35].

### 3.1. Implications of FoxO in inflammatory response

**Figure 1.**
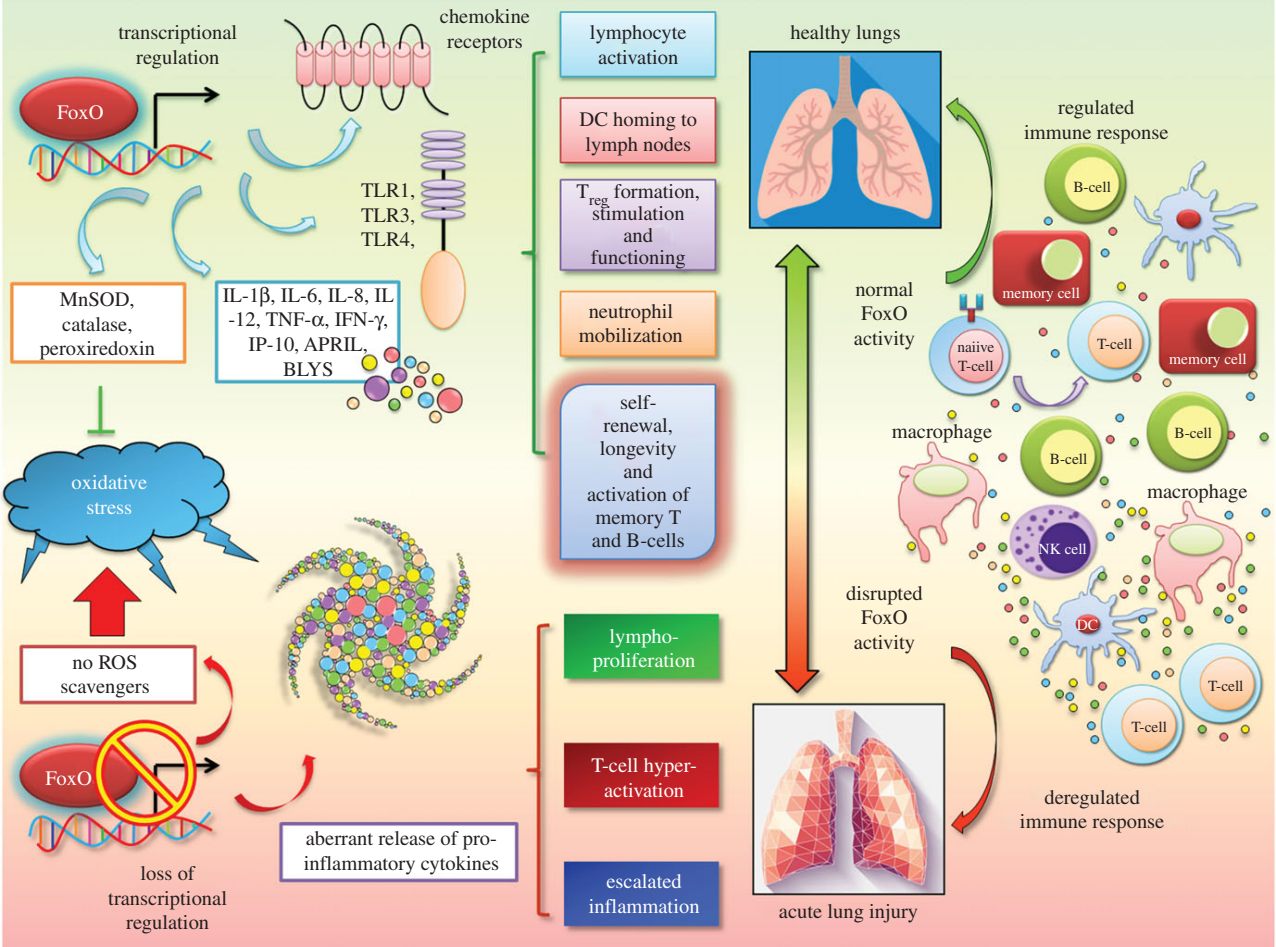
FoxOs are unique master regulators of cellular inflammatory and redox responses. The FoxO family of transcription factors modulates transcriptional expression of numerous genes that are part of the host inflammation mechanism, including several interleukins, chemokines and cytokines. FoxOs also play pivotal roles in the redox homeostasis and immune response. The loss of this intricate regulatory mode of FoxOs may, thus, lead to inefficient oxidative stress response, abnormal immune cell activation and a state of exacerbated inflammation, reminiscent of a ‘cytokine storm’.

FoxO proteins are crucial players for both the execution and resolution of inflammation. This is achieved via dynamic transcriptional regulation of the expression of multiple classical inflammatory factors in a context- dependent manner (figure 1). Previous studies have suggested FoxO factors as prime regulators of the innate immune system [7]. This is exemplified in the management of inflammation by FoxOs through escalated TLR3/4- mediated signalling and IL-1β expression in human macrophages [6]. FoxO1, in particular, stimulates transcriptional expression of pro-inflammatory molecules like TLR1 and 4, IL-1β and TNF-α, chemokine receptors, namely C–C chemokine receptor type 7 (CCR7) and C–X–C chemokine receptor type 2 (CXCR2), B-cell modulators such as (A proliferation inducing ligand) APRIL and (B lymphocyte stimulator) BLYS, T-cell regulators, including (cytotoxic T-lymphocyte-associated protein 4) CTLA4, in addition to anti-oxidants. FoxO1 is also an essential requirement for T-cell tolerance and naive T-cell homeostasis, homing of dendritic cells and B cells and initiation of an adaptive immune response to bacterial challenge [3,9]. Furthermore, FoxO1 serves in transcriptional modulation of IL-9-generating T helper (T_h_) 9 cells that participate in inducing immunity against extracellular pathogens. Intriguingly, pulmonary overexpression of IL-9 has been demonstrated to play a role in lymphocytic and eosinophilic inflammatory infiltration, mast cell hyperplasia and mucus secretion [36].

Notably, FoxO3 expression is found within the airway epithelium apart from macrophages and other cell types in the lungs [37,38]. FoxO3 plays an extremely important role in overseeing innate immune responses to infections faced by the airway epithelium. In response to bacterial challenge in bronchial epithelial cells, FoxO3 led to the expression of antimicrobial factors like human β-defensin 2 and various cytokines, including IL-6, IL-8, TNF-α, C–X–C motif chemokine ligand 10 (CXCL10) [6].

Contemporary studies have unveiled that inhibitory phosphorylation of FoxOs in different immune cells like macrophages and lymphocytes are associated with inflammatory cell activation in rheumatoid arthritis and osteoarthritis patients. Complementary to such observations, certain clinical works have demonstrated suppressed *foxo1* gene transcript levels in individuals afflicted with systemic lupus erythematosus and rheumatoid arthritis. Such results illustrate a potential contribution of FoxO deregulation as an aetiology for these inflammatory disorders [39].

Numerous studies illuminated the role of the FoxO family members as both sensors of oxidative stress signals as well as regulators of the subsequent cellular response. The transcriptional network downstream of these redox sensitive proteins is dependent, at least in part, on the oxidative status. Kops *et al*. [40] demonstrated that FoxO1 and FoxO3 facilitated the synthesis of ROS-scavenging enzymes, such as manganese superoxide dismutase (MnSOD) and catalase in response to oxidative stress within the cell. Furthermore, FoxO3 enhanced the activity of peroxiredoxin III (Prx III), another endogenous anti-oxidant [41]. FoxO3 activation resulted in destabilization of hypoxia-inducible factor (HIF) 1-α, thus curbing hypoxia-mediated increase in ROS [42]. Clearly, numerous evidences point to the fact that insufficient FoxO activity may cause elevated cellular damage in the presence of large concentrations of ROS. Of note, excessive oxidative stress is known to reduce type I and type III IFN responses to viral infection in airway epithelial cells [43].

Moreover, strong nuclear staining of FoxO3 is witnessed in the lungs of patients with various infection-related lung disorders such as cystic fibrosis, chronic obstructive pulmonary disease and severe pneumonia with acute respiratory distress. In such individuals, FoxO3 level was negatively correlated with IL-8 generation in the airway epithelial cells [37]. Also, FoxO3 thwarted oxidative stress and thereby repressed lung inflammation in mice exposed to cigarette smoke [38]. Such results clearly revealed the contribution of FoxO3 to both modulation of antiviral responses and inhibition of pro-inflammatory chemokine expression. Thus, activation of FoxO3 potentially alleviates the expression of pro-inflammatory cytokines in response to viral infection, thus imparting protection against lung inflammation. Lin *et al*. [44] showed that the ablation of FoxO3 may lead to spontaneous lympho-proliferation, T-cell hyper-activation and escalated inflammation with a pronounced rise in the levels of inflammation favouring molecules like NF-κB, IL-2 and IFN-γ. Whether SARS-CoV-2 abrogates the regular functions of FoxOs to execute pulmonary distress and whether restoring FoxO activity may resolve the cytokine storm are highly possible and intriguing theories that remain to be tested.

### 3.2. FoxO partakes in viral infection

The FoxO signalling pathway is significantly implicated in manipulating the anti-apoptotic and anti-inflammatory responses during influenza virus A-induced respiratory pathogenesis [45]. Wu *et al*. suggested that sustaining a normal level and function of FoxO1 were consistent with the inhibition of the pro-apoptotic effects of influenza virus infection. FoxO1 has been identified as a negative regulator of cellular antiviral response triggered by exposure to virus. FoxO1 suppression was accompanied by the activation of virus-induced interferon-stimulated response element (ISRE) as well as IFN-β production [46]. Importantly, FoxO3 has been shown to regulate antiviral responses. The zebrafish *foxo3b*, an orthologue of mammalian FoxO3, interacted with and transcriptionally disrupted interferon regulatory factor (IRF) 3/7 activity. This led to a strong repression of IFN activation following viral infection. Thus, targeted inhibition of critical antiviral genes was a key mechanism adopted by *foxo3b* to negatively regulate cellular antiviral response [47]. It is well known that IRF7 partners with IRF3 to modulate the type I IFN response in mammalian viral infections. The FoxO signalling pathway was determined to be an essential immune-modulatory cascade that was regulated by IRF7 as a functional host immune response to viral infection [48]. In line with the role of FoxOs in cellular antiviral response, they were found to impart negative regulation of IRF. FoxO1 was associated with ubiquitin-tagged proteasomal degradation of IRF3 [46] while FoxO3 directly suppressed IRF7 transcription in mice macrophages [49]. This regulatory circuit can prevent excessive innate immune response, which may have pathological outcomes. Mammalian FoxO3 plays a critical role in mediating antiviral type I and type III IFN responses to clear rhinovirus [50]. In agreement, FoxO3 deficiency in these cells led to mitigated IFN response to rhinovirus infection. The knockout of FoxO3 in virus-infected mice resulted in diminished levels of IFN-α, IFN-β and IFN-λ. In addition, these mice displayed persistent viral load, increased lung inflammation as well as heightened production of pro-inflammatory cytokines. Pre-treatment with specific anti-oxidants was found to rescue antiviral IFN responses in the knockout cells. Suppression of oxidative stress also abolished the enhanced pro-inflammatory cytokine responses to viral infection [50]. Therefore, FoxO3 is indispensible for mediating antiviral responses apart from imparting protection against hyper-inflammatory cytokine response.

## 4. Cellular FoxO is exploited in SARS-CoV-2 infection

As has been stated, interaction of the host ACE2 with SARS-CoV-2 spike protein is the key determinant for viral invasion. Although the application of ACE inhibitors/angiotensin-receptor blockers is increasingly being considered for COVID-19, such molecules may unfavourably alter the balance between ACE and ACE2. This will only boost the number of docking sites for viral entry [51,52]. Initial clinical trials demonstrated a dramatic inhibition of viral propagation in the presence of a human recombinant soluble ACE2 *in vitro*. However, extensive validation is warranted for determining the clinical efficacy of such an approach [53]. The characterization of the ACE2 promoter regions revealed that the lungs exhibit expression primarily from the distal promoter region. A functional binding site for forkhead box transcription factors was identified as a novel and important *cis*-regulatory element that affected the expression of ACE2. FoxO1 was recognized to bind to the region spanning 2153/2144 nucleotides and its expression was correlated with ACE2 transcript level to some extent [54], implying that FoxO factors may be tweaked to modify ACE2 availability during SARS-CoV-2 entry into the cell.

The expression of ACE2 is commonly increased in the case of cellular stress. This may include hypoxia, IL-1β treatment or exposure to the antimicrobial peptide (AMP) mimic 5-amino-4-imidazole carboxamide riboside (AICAR). IL-1β is the initial responder to impart protective effects and stimulate epithelial repair in acute respiratory distress situations [55,56]. The silent information regulator T1 (SIRT1) deacetylase was found to bind to the ACE2 promoter. This interaction was altered by both AICAR and IL-1β treatments. Importantly, the loss of SIRT1 activity ablated the AICAR-mediated increase in ACE2 level. The data thus established SIRT1 as a primary transcriptional regulator of ACE2 expression, particularly in situations of energy stress [57]. What is exciting is that SIRT1 has been shown to exert the majority of its protective effects via deacetylation of its target proteins, a distinctive example being FoxO factors [58]. These results suggest that FoxO may be a crucial link between IL-1β and SIRT1 regulated expression of ACE2. This further substantiated our previous suggestion to exogenously modulate FoxO for minimal ACE2 accessibility to SARS-CoV-2.

Similar to other coronaviruses, SARS-CoV-2 may exploit the host translation machinery to advance the production of its own components [59]. Inactivation of eukaryotic initiation factor 2 (eIF2) is expected to be a part of the host countermeasure to SARS-CoV-2 infection [60]. Incidentally, SIRT1 was upregulated in the lungs of COVID-19 patients exhibiting severe symptoms [61]. SIRT1 has been found to monitor the negative feedback regulation of eIF2α phosphorylation. The loss of SIRT1, in fact, led to constitutive phosphorylation of eIF2α, although its downstream signalling was delayed and suppressed [62]. This was indicative of a weaker translation recovery post-stress and established SIRT1 as a critical mediator of eIF2α-associated integrated stress response. As a result, ongoing research has been centred on validating SIRT1 inhibitors for treating COVID-19 [63]. It is worth noting that conventional SIRT1 inhibitors, such as nicotinamide, curbed the activity of FoxO members to exert their downstream effects [64]. The phosphorylation of eIF2α by protein kinase R (PKR) and PKR-like endoplasmic reticulum kinase (PERK) inhibits protein synthesis. SARS-CoV infection triggered PERK activation within the host cells [65]. Of note, PERK reportedly phosphorylates and potentiates FoxO activity, whereas PERK depletion imparted a reverse effect [66]. More recently, PERK was discovered as a novel target of FoxO3 with a positive correlation existing between their expressions. FoxO3 expression was also significantly connected to PERK–eIF2α pathway activation [66,67]. These studies clearly unveil that, analogous to other viruses, SARS-CoV-2 possibly hijacks the FoxO regulatory circuit for pathogenesis and incline towards the potential of modulating host FoxO proteins in impeding SARS-CoV-2-related complications.

Emerging evidence points to the role played by FoxO3 in directing transcriptional regulation of Kelch-like ECH-associated protein 1 (Keap1). Keap1 acts as an adaptor protein that targets the multifaceted nuclear factor erythroid-2-related factor (Nrf2) for ubiquitin-mediated degradation [68]. Nrf2 is considered as a master modulator of anti-oxidant and anti-inflammatory responses [69,70]. The active cross-talk between FoxO3 and Nrf2 pathways impacts diverse cellular responses, such as proliferation, survival and oxidative defence [71]. SARS-CoV-2 was recently identified to inhibit Nrf2 to rid the infected cells of a critical cytoprotective signalling mode [72]. Considering the remarkable protective features characteristic of the FoxO family, it can be put forward that SARS-CoV-2 may try to deprive the host cell of the FoxO signalling network as well to favour viral growth. Further investigation of this aspect may unravel a unique mechanism underlying COVID-19 pathogenesis through modulation of FoxO functions.

In addition, enhancement of the FoxO transcriptional target, haem oxygenase 1 (HO-1) [73], has been connected to antiviral responses against a plethora of viruses, including human immunodeficiency virus, hepatitis virus, influenza virus and respiratory syncytial virus, among others [74]. HO-1 majorly induces its antiviral response through a heterodimeric complex formed with IRF3, eventually leading to expression of type I IFNs [75]. This putative anti-inflammatory enzyme catalyses the degradation of free haem into biliverdin/bilirubin, iron/ferritin and carbon monoxide. Free haem is currently postulated to stimulate several of the inflammatory features observed in critical COVID-19 patients while its degradation products antagonized SARS-CoV-2 activity [76]. In agreement, subjects characterized by old age, some kind of metabolic syndrome and decreased level of HO-1 were found to be more vulnerable to the risk of COVID-19 infection [77]. Hence, induction of HO-1 directly or via FoxO activation is postulated to impart protection and is expected to mitigate SARS-CoV-2 infection, thus protecting the lungs from inflammatory assault.

Infection with SARS-CoV has been shown to elicit nuclear factor κ B (NF-κB) activation in mice lungs and human monocyte macrophages while NF-κB suppression led to the ebbing of inflammation and improved survival rate in infected mice [78,79]. Interestingly, FoxO3 has been corroborated as a negative regulator of NF-κB signalling. A reciprocal correlation exists between the expression and activities of FoxO3 and NF-κB in immune cells. Functional studies have further revealed that the absence of functional FoxO3 led to spontaneous increase in NF-κB activation in addition to hyper-activation of CD4^+^ T cells and a robust multi-systemic inflammatory syndrome [44]. Lee *et al*. showed that FoxO3 upregulation induced the expression of *κ*B-Ras1, which inhibited NF-κB activation [80]. Furthermore, SIRT1 was also found to antagonize the NF-κB pathway [81]. Given the indispensible involvement of FoxO in SIRT1 functions, it is only reasonable to propose that FoxOs play a distinguished role in this inflammation countering mechanism of SIRT1. Taken together, pharmacological activation of FoxO factors can effectively prove to be an attractive strategy to limit inflammation inflicted by NF-κB in the lungs of SARS-CoV-2-infected individuals.

The activation of AMPs is an important type of immune effector response to fight pathogenic infections. Lactoferrin is an AMP that has been demonstrated to display potent antiviral properties against SARS-CoV infection [82]. Contemporary scientists are, therefore, attempting to use AMPs like Lactoferrin as an adjuvant therapy for the management of COVID-19 [83]. Of significance, AMP induction is elevated in the presence of FoxO overexpression while it is abrogated in *foxo* null mutants. The loss and gain of functions data revealed a prominent role of nuclear FoxOs in the activation of AMP genes through direct transcriptional regulation of the latter and this mechanism is evolutionarily conserved. Notably, in animals who fail to respond to immune challenges owing to defects in the Toll and immune deficiency pathways, this FoxO-dependent mode of action ensured the production of AMPs to maintain and strengthen the defence barrier [7]. Combination of such AMP-related antiviral regime with FoxO activation strategy may naturally provide a superior protection for combating the dangerous COVID-19 infection.

As has been discussed earlier, FoxOs negatively impact the activation of IRFs and the subsequent IFN response. FoxO3 silencing reportedly stifled IFN-γ-associated major histocompatibility complex (MHC) II expression whereas FoxO3 upregulation stimulated cytotoxic *trans*-activator (CIITA)-induced *trans*-activation of the MHC II promoter [84]. Since CIITA has been implicated in resisting the endosomal entry of SARS-CoV-2, modulation of the FoxO3- CIITA signalling is likely to aid in managing the viral infection [85]. In line with such studies, pulmonary alveolar cells or resident macrophages infected with SARS-CoV-2 released excessive IFNs, which exacerbated the pulmonary inflammatory damage [86]. This evidently reflects that SARS-CoV-2 may abrogate normal FoxO regulation of IFNs, which culminates in the hyper-inflammation. MERS, SARS and few other coronaviruses have been revealed to be prominently susceptible to IFN treatment [87]. If this mechanism is also mirrored in the case of COVID-19, pharmacological modulation of the FoxO family constituents holds great promise to mitigate the IFN-mediated inflammatory response confronted in such patients. In fact, the use of recombinant IFNs have begun to inhibit SARS-CoV-2 replication and protein synthesis (NCT04293887), corroborating our notion [88].

The T_h_2, T_h_9 and T_h_17 cells that produce a generous amount of IL-9 are irrevocably vital for allergic inflammatory response, autoimmune syndrome and immunity to pathogenic invasion [89]. FoxO1 has been recognized as the critical transcription factor necessary for IL-9 induction in these immune cells. Mechanistic insights indicated that FoxO1 *trans*-activated IL-9 in these T cells. This is the primary mechanism deployed by FoxO1 to ameliorate allergic inflammation as seen in asthma [8]. The suppression of disease phenotype in lupus mice models was possible by the inhibitory peptide that acted via FoxO3 while FoxO1 level correlated with disease severity in several lupus patients. Furthermore, in mice with multiple sclerosis, pro-inflammatory factors contributed to auto- aggressive T-cell activity through FoxO3 inhibition. Importantly, FoxO1 drove the production of IL-10 cytokine by transcriptionally monitoring its expression [90]. IL-10 is particularly upregulated in SARS-CoV-2 patients by an appreciable extent. Taking into account the vital contribution of FoxO factors in keeping a check on excessive inflammation, it may be logical to imply its clinical significance in fighting the cytokine storm found in COVID-19 persons.

Although the number of clinical studies is limiting, scientists believe that there is a clear correlation between oxidative stress and the severity of COVID-19 disease. Many lines of evidence in preclinical settings suggest that ROS elevation coupled to a deprived anti-oxidant system markedly encouraged the progression of SARS-CoV pathogenesis. The onset of lung injury in infected patients has been shown to rely on the oxidative stress machinery, which is generally linked with activation of transcription factors, including NF-κB. This elicits the aggravated pro- inflammatory host response [91]. Considering that FoxO factors prominently influence the cellular anti-oxidant mechanisms, we suggest that ROS-activated FoxO transcriptional cascade may be a key player in SARS-CoV-2 pathophysiology. The TLR4 signalling pathway has also been recently recognized as a vital mechanism that can mediate the severity of acute lung injury [92]. Oxidized phospholipids, generated by macrophages in human lungs infected with the SARS virus, led to the activation of the TLR4 circuit. This evidently induced cytokine over- production and lung injury [91]. We have already mentioned that FoxOs are primary regulators of the TRL4 signalling, thereby implying the therapeutic potential of FoxO modulation in TLR4 response in COVID-19 patients.

**Figure 2.**
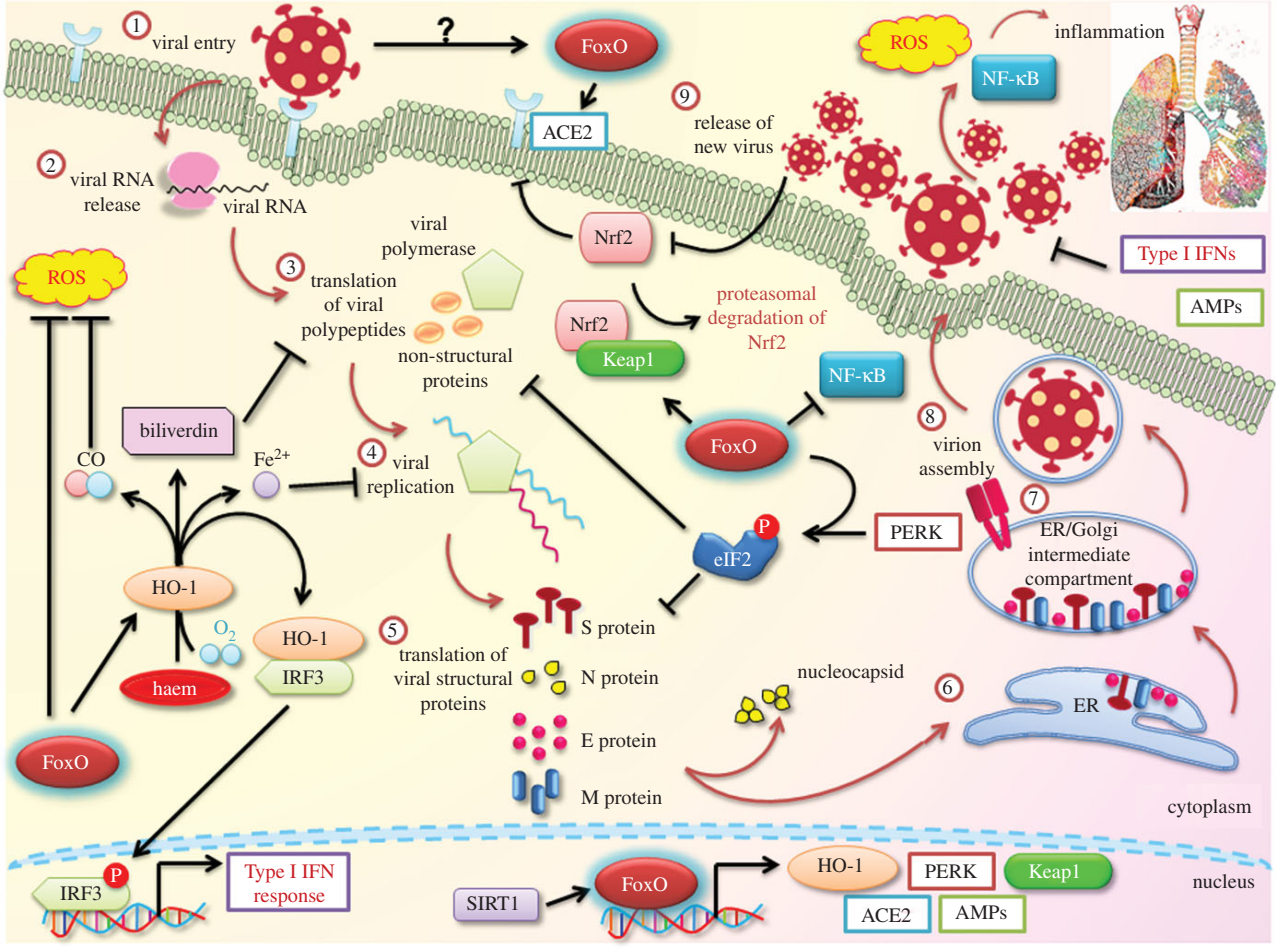
Potential involvement of FoxO and its downstream transcriptional network in SARS-CoV-2 life cycle. The SARS-CoV-2 viral life cycle is initiated by viral recognition by host ACE2, which facilitates viral entry. Following uncoating of the viral nucleocapsid in the cytoplasm, translation of the viral RNA and subsequent cleavage generates specific viral proteins. Next, viral replication and generation of crucial viral structural components take place. Finally, these are assembled in the ER and the ER/Golgi complex and new virions are released out of the cell. These go on to freshly infect pulmonary cells and induce inflammatory shock and oxidative stress. Host defence is mediated by the inhibition of the protein translational machinery. SARS-CoV-2 infection activates PERK, which phosphorylates eIF2 and represses protein translation. FoxOs actively aid in this phosphorylation step. Also, ACE2, HO-1, PERK, AMPs and Keap1 are few of the relevant transcriptional targets of FoxO transcription factors. HO-1 catalyses haem into carbon monoxide (CO), biliverdin and Fe^2+^. The latter two factors interrupt different steps of the SARS-CoV-2 life cycle while CO antagonizes ROS stress. HO-1, via type I IFNs, and AMPs also assist in mediating antiviral response. Keap1 leads to Nrf2 degradation and recent studies have implied Nrf2 involvement in COVID-19. Another essential pro- inflammatory molecule suppressed by FoxOs is NF-κB, which is exploited by SARS-CoV-2 for imparting inflammation-associated lung injury. FoxOs themselves are regulated by SIRTs, which are currently being explored for their role in COVID-19 pathogenesis. Therefore, SARS-CoV-2 is likely to hijack the FoxO network to deregulate its effector response and establish infection.

As depicted in figure 2, there exists a strong possibility that SARS-CoV-2 may hijack and promote dysregulation of the FoxO factors for carrying out its infectious cycle. An interesting study in canine coronavirus type II demonstrated that FoxOs were responsible for influencing the pro-apoptotic effects of the virus, thereby showing a critical involvement of FoxOs in this infection. This was, in part, mediated through the regulation of TNF-related apoptosis-inducing ligand (TRAIL), Bcl2 and Fas/FasL levels [93]. This study paved the way for the direct functional relationship between the FoxO family proteins with a prominent member of the Coronaviranae family.

## 5. FoxO governs host immune response: a breakthrough in managing COVID-19?

Memory T cells induced by previous pathogenic challenges are unequivocally relevant for protection against subsequent infections, as has been reported for influenza virus H1N1 strain [94]. Even though most acute infections give rise to such a protective immunity to fight off future viral exposure, collation of evidence related to human coronaviruses suggests a contrarian view. This implies that individuals infected with this family of viruses may never benefit from the extensive protection provided by the adaptive immune response [95–97]. Several possible factors own up to this malfunction, including insufficient T/B-cell response. A milestone study back in 2014 had emphasized on the lack of appropriate neutralizing antibody titres and the extremely short-lived memory B-cell response in SARS-recovered individuals [98]. Initial screening had recognized that SARS-CoV-specific antibody is quite unstable; the IgA and IgM response to this virus failed to last for more than six months. The presence of memory T cells in individuals following respiratory coronavirus infection has been shown by several groups, albeit their *in vivo* efficacy in virus clearance is a poorly researched area [99]. SARS-CoV-infected population demonstrated a clear dysregulation in the CD4 and CD8 T-cell activation in addition to a delayed and impaired adaptive immune response. The infection also abrogated dendritic cell migration, which resulted in weakened homing of T cells and reduced virus-specific T-cell production [98]. The same report had shown, particularly for aged models, that SARS-CoV infection was associated with a considerable decrease in the virus-specific CD8^+^ T cells within the lungs. Inadequate T-cell innate immunity could explain the exuberated innate response and the underlying viral pathogenesis, especially in aged patients as witnessed for SARS-CoV-2 pandemic. Late T-cell responses usually climax in an amplified inflammation in the presence of a viral infection, which has been re- iterated as the primary stimulant for the extreme pulmonary distress in COVID-19 patients [100].

Immunity following chronic viral infections relies on the maintenance of antigen-specific CD8^+^ T cells, although the transcriptional requirements of these cells are still not fully elucidated. A diverse array of studies has pointed towards the essential role of FoxO factors in regulating specialized lymphocyte functions. FoxO1 inactivation directs the homeostasis of CD4 conventional and regulatory T (T_reg_) cells wherein enforced expression of FoxO1 inadvertently hampers this equipoise [101]. The abrogation of FoxO expression was also linked to progressive drop in the frequency of T_reg_ cells in the peripheral tissues and their immune-suppressive capacity was found to be significantly hampered, thus emphasizing the importance of FoxO expression in maintaining immunological tolerance [102]. T_reg_ cells specifically depleted of FoxO1 produced more IFN-γ when compared with wild-type cells [103]. This was in sync with previous observations of IFN suppression by FoxO proteins. Utzschneider *et al*. [104] found that the continued expression of FoxO1 is an indispensible necessity for preserving the longevity, self- renewal and the ability to shift between quiescence and cell division of CD8^+^ memory T-cell population. Inactivation of *foxo1* gene led to the reversion of memory T cells to a state of terminal differentiation, which prevented a secondary memory response in multiple cases of infection [10]. The deletion of *foxo1* after the clearance of an infection resulted in a rapid loss of typical gene expression patterns (almost 90%) of memory T cells. Even during a persisting viral infection, the depletion of FoxO1 activity caused a dramatic decline of T-cell expansion while giving rise to T cells deficient in effector cytokines and exhibiting features of anergy [104–106]. This underscored the broad importance of FoxO1 in manifesting the post-effector immune programme, a prerequisite for forming the long-lived memory of T cells. FoxO1 is additionally important for proliferation, differentiation, survival and class switching in B cells. FoxO1 has been shown to direct the development of germinal centres, which are a necessity in development of clonal variants of B cells. The depletion of *foxo1* in germinal centre B cells led to diminished somatic hyper-mutation and dwindled class switching, which significantly hampered a robust antibody response towards infections [107]. The loss of *foxo1* in dendritic cells resulted in reduction of multiple phenomena, such as cytokine production, homing of dendritic cells to the lymph nodes, activation of CD4^+^ T and B cells and antibody generation, thereby enhancing sensitivity to pathogenic challenges [108]. FoxO3 was also identified as a prime modulator of CD8 T-cell memory while FoxO3 therapeutic modifications have been proposed to convalesce protective immunity to ward off intracellular pathogens [109]. A deficiency in FoxO3, following a viral infection, was shown to facilitate considerably exaggerated expansion of T-cell populations. This arose from dendritic cell-specific rise in the production of IL-6. This caused variations in the stimulatory capacity of FoxO3-deficient dendritic cells to sustain T-cell viability. The use of CTLA4 led to FoxO3 nuclear localization, which consequently suppressed heightened release of IL-6 and TNF. Such data inclined towards the contribution of FoxO3 in constraining production of key inflammatory cytokines and controlling T-cell viability [110]. Instead of eliminating the virus-infected cells, a dysfunctional T-cell response owing to abrogated FoxO function may manifest in the loss of immune regulation and favour progression of COVID-19 disease. Since the FoxO factors are intimately involved in overseeing both the innate and humoral immune responses, their participation during SARS-CoV-2 host response is inevitable and necessitate future interrogation. The generation of surplus T cells specific for SARS-CoV-2 within the necessary time frame may prove to be the most competent strategy to withstand viral infection. Hence, modifying FoxO transcription factor is likely to evoke the naive T and B cells for proliferation and differentiation in conjunction with immune reactivation, which may be one of the most lucrative modules for resisting COVID-19.

## 6. Pharmacological modulation of FoxO activity as potential anti-SARS-CoV-2 therapy

In the wake of the current pandemic, the global scientific community has been exploring every possible opportunity to develop a curative or palliative strategy against coronavirus. Clinicians have been trying to manage the aggravated inflammation observed in COVID-19 patients using an armamentarium of anti-inflammatory drugs and molecules that target cytokines. Such practices are at an early stage, and therefore not entirely conclusive. For example, dexamethasone at a dose of 6 mg daily for 10 days decreased mortality in specific COVID-19 patients in a context-dependent manner [111]. However, dexamethasone is also known to stall the functions of immune T and B cells in the host body, thus restricting its current use exclusively in severely infected and intubated patients [112]. The possible repurposing of tocilizumab, a humanized antibody against IL-6 receptor, and anakinra, a recombinant IL-1 receptor agonist, for COVID-19 therapy has been under intensive research [113,114] while the IL-6 antagonist sarilumab (NCT04315298), the TNF-α suppressor thalidomide (NCT04273529, NCT04273581) and methylprednisolone (NCT04273321, NCT04263402) are notable examples of anti-inflammatory pharmaceuticals undergoing trials for their potent applications in severe SARS-CoV-2 infections. Similarly, based on a recent comparative cohort study, impeding the IL-17 pathway has also been suggested as a potential strategy to combat COVID-19 infection [115].

**Figure 3.**
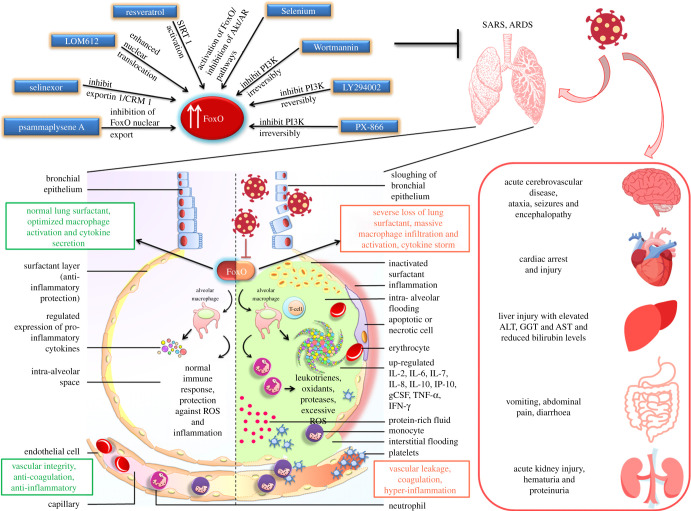
Pharmacological restoration of FoxO activity potentially mitigates COVID-19 pathophysiology. SARS-CoV-2 induces severe acute respiratory syndrome (SARS) and acute respiratory distress syndrome (ARDS) within the lungs and, eventually, causes multi-organ failure all across the body. This shuts down almost every host system and culminates in death in severely infected patients. FoxOs are vital regulators for imparting cytoprotection and anti-inflammation. Pharmacological modulation of the deregulated FoxOs within the SARS-CoV-2-infected cells may restore normal protective functions of FoxOs and help resolve COVID-19 symptoms and the systemic failure.

In this context, enhancing the activity of FoxOs can prove immensely valuable as it will not only assist in curtailing the cytokine storm in the patients, which is the major cause of COVID-19-related mortality, but also bolster the immune response and foster the retention of immunological memory against the infection (figure 3). Cautain *et al*. [116] reported the discovery of a small molecule activator of FoxO, a novel isothiocyanate, LOM612, which was shown to enhance the levels of FoxO1 as well as FoxO3 in the cell nucleus by augmenting their nuclear import in a dose-dependent manner. Furthermore, inhibitors of Exportin 1 have been documented to promote FoxO1 activity by a similar mechanism [117]. A small FoxO1-derived peptide, FOI-6nls, disturbs its phosphorylation by CDK1/2 and subsequently stimulates its activity [118]. Each of these FoxO-activating agents holds vast potential in mitigating the exaggerated inflammation related to SARS-CoV-2 infection while re-directing the immune system for viral clearance. Hence, these molecules can be immediately expedited to relevant clinical trial to determine their efficacy in SARS-CoV-2-infected patients for their repurposing as an anti-COVID-19 therapeutic regime (table 1). On the same note, since the PI3K/Akt pathway acts as a major upstream regulator of FoxO levels, inhibiting PI3K and/or Akt can serve as a crucial tool in fighting the cytokine downpour in COVID-19 patients.

**Table 1.** List of compounds reported to upregulate levels of FoxO transcription factors.

s.no.	compound name	mol. wt. (g mol^−1^)	mode of action	structure	reference
1.	resveratrol	228.24	activates SIRT1 thus enhancing FoxO deacetylation	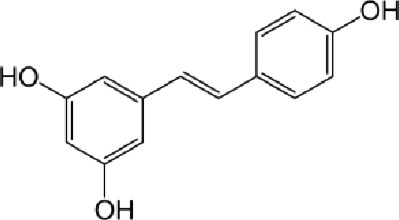	[64]
2.	LOM612	258.3	enhances FoxO nuclear translocation	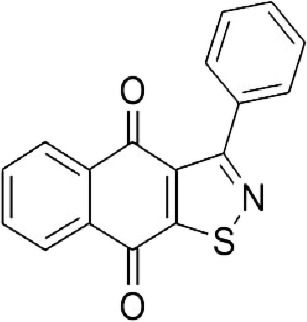	[116]
3.	selenium	78.96	direct activation of FoxO or through inhibition of Androgen receptor (AR) and/or Akt pathway	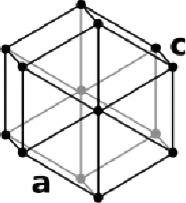	[119]
4.	psammaplysene A	769.2	inhibits nuclear export of FoxO	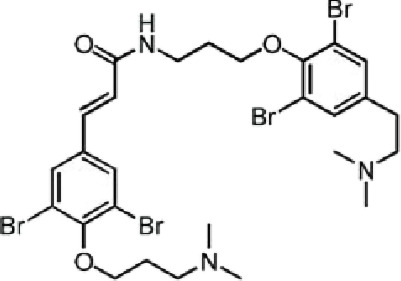	[120]
5.	selinexor (KT-330)	443.3	inhibits Exportin1 (XPO1)/Chromosome maintenance region1 (CRM1)	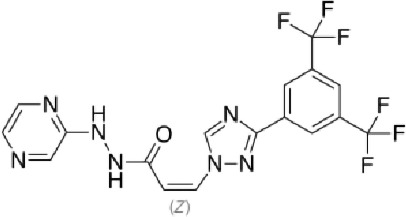	[121]
6.	wortmannin	428.4	inhibits PI3Ks irreversibly	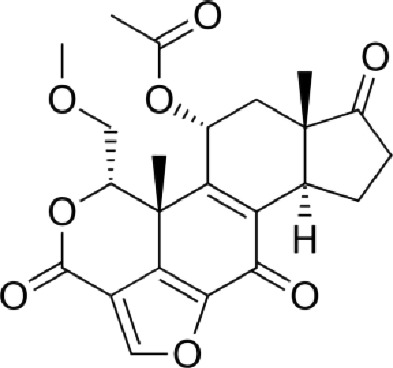	[122]
7.	LY294002	307.349	inhibits PI3Ks reversibly	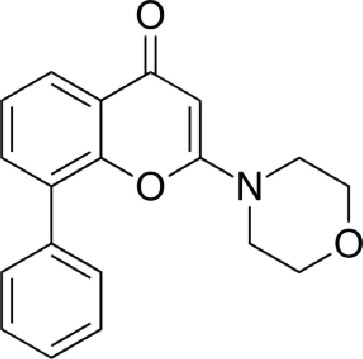	[123]
8.	PX-866	525.6	inhibits PI3Ks irreversibly	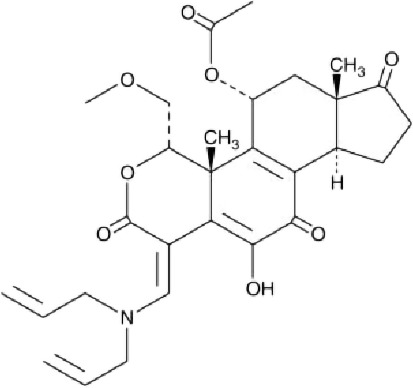	[124]

Overall, the amalgamation of FoxO activation and anti-inflammatory impact of such compounds is strongly believed to provide a robust cytoprotection and warrants intensive exploration for efficient management of COVID-19 symptoms.

## 7. Conclusion and future directions

Despite extraordinary efforts over the last one year, currently we do not have any effective treatment for this inexplicably evolving deadly pandemic. While few vaccines have been approved for emergency application to curb the rapid spread of the pandemic, these approaches have far to go before their long-term efficacy and possible side effects can be evaluated (reviewed in [125]). Since these vaccines have been fast-tracked for commercial use with limited clinical trial data, their extent of protection against the virus is still dubious [125]. Moreover, given the infrastructural and financial challenges, only a minor percentage of the population is getting vaccinated at a given time. This means that by the time the entire global population gets vaccinated, the initial epitope configuration of the virus will have mutated by several folds. With the surge in genotypic variants of SARS-CoV-2, the efficacy of currently employed vaccines, especially the threshold of immune response elicited in varied demographic cohorts, needs to be closely monitored [126,127]. Furthermore, vaccines are agents to prevent the disease but not cure it; hence, potential alternatives for treating patients are an imminent requirement. Therefore, a growing number of studies has been focused on repurposing existing therapeutic agents with effective antiviral and anti-inflammatory properties as a rapid measure to control this fatal pandemic. These agents may be aimed at either directly inhibiting the viral survival pathways or eliciting the antiviral innate immunity or assuaging the damage provoked by an out-of-control cellular inflammation. Remdesivir and Favipiravir are by far the most attractive candidate molecules that have been found to improve the condition in COVID-19 patients with minimal adverse side effects [128]. Both these agents interrupt the RNA-dependent RNA polymerization and halt viral replication. Therefore, contemporary investigations are focused on the integration of more systematic approaches to distinguish the most prospective agents for drug repurposing in the battle against this global pandemic.

Our review is an attempt to propose pharmacological activation of the FoxO transcription factor as a potent mechanism to tackle the deadly manifestation of SARS-CoV-2. Infection with SARS-CoV-2 is usually accompanied by a dramatic reduction in anti-atrophic, anti-inflammatory, anti-fibrotic and anti-oxidative responses, which contribute to acute lung injury. We envisage that the activation of the FoxO regulators can successfully keep the host inflammatory response in check to prevent the uncontrolled burst of pro-inflammatory molecules as well as impart a level of protection to the cells against viral assault and aid in repair of the damaged pulmonary tissue. Such anti-oxidant and pro-protective impacts of pharmacological restoration of FoxO are expected to persist longer than conventional treatment modalities (such as vitamin C) [129]. Infection with SARS-CoV-2 often snowballs into multiple organ dysfunction, resulting in a high mortality rate, and in this regard the role of host inflammatory response has been thoroughly questioned. Most strikingly, the involvement of FoxO proteins in mediating the host infection tolerance mechanisms has garnered attention. FoxO is a pivotal cell signalling mediator that controls the balance of damage and repair processes, which eventually dictates the fate of the affected organs and, in turn, the host. Our literature survey has strongly established FoxO as a potential cellular factor that may be targeted by SARS-CoV-2 in order to sustain its infective mode. Therapeutic targeting of FoxO may be a reasonable approach to limit the domino-like severe damage on the multiple organs imparted by COVID-19 infection and may offer novel alternatives beyond currently used strictly supportive therapies (figure 3). Nonetheless, such manipulation of multifunctional cellular signal regulators is likely to generate undesirable cell-type-dependent effects. Different modes of targeted delivery might help to resolve such hurdles. Future investigation should focus on unravelling the mechanism by which SARS-CoV-2 potentially modulates host FoxO so that each point of interaction may be targeted for overall protection. Whether FoxO targeting has any impact on viral entry, replication and survival, and which downstream effectors of FoxO signalling may be involved in this host–pathogen network, are critical areas for further study.

Nevertheless, advanced clinical validation can provide a clear route to the efficacy of FoxO activators, direct or indirect, in COVID-19-infected individuals. If proved successful, this regime can be rapidly mobilized to enhance recovery of COVID-19 patients. Finally, this wealth of information should also lay the groundwork to stay prepared with an arsenal of potentially repurposed molecules for possible future viral outbreaks.

## Data accessibility

This article has no additional data.

## References

[1] Ren L-L et al. 2020 Identification of a novel coronavirus causing severe pneumonia in human: a descriptive study. Chin. Med. J. *(*Engl) **133**, 1015-1024. 32004165 10.1097/CM9.0000000000000722 PMC7147275

[2] Flahault A. 2020 Has China faced only a herald wave of SARS-CoV-2? Lancet **395**, 947. (10.1016/S0140-6736(20)30521-3) PMC7124610 32145187

[3] Wang Y, Zhou Y, Graves DT. 2014 FOXO transcription factors: their clinical significance and regulation. Biomed. Res. Int. **2014**, 1-13. 10.1155/2014/925350 PMC4016844 24864265

[4] Al-Tamari HM et al. 2018 FoxO3 an important player in fibrogenesis and therapeutic target for idiopathic pulmonary fibrosis. EMBO Mol. Med. **10**, 276-293. (10.15252/emmm.201606261) 29217661 PMC5801513

[5] Nho RS, Hergert P, Kahm J, Jessurun J, Henke C. 2011 Pathological alteration of FoxO3a activity promotes idiopathic pulmonary fibrosis fibroblast proliferation on type I collagen matrix. Am. J. Pathol. **179**, 2420-2430. (10.1016/j.ajpath.2011.07.020) 21893017 PMC3204034

[6] Seiler F et al. 2013 FOXO transcription factors regulate innate immune mechanisms in respiratory epithelial cells. J. Immunol. **190**, 1603-1613. (10.4049/jimmunol.1200596) 23315071

[7] Becker T, Loch G, Beyer M, Zinke I, Aschenbrenner AC, Carrera P, Inhester T, Schultze JL, Hoch M. 2010 FOXO-dependent regulation of innate immune homeostasis. Nature **463**, 369-373. (10.1038/nature08698) 20090753

[8] Malik S et al. 2017 Transcription factor Foxo1 is essential for IL-9 induction in T helper cells. Nat. Commun. **8**, 1-14. (10.1038/s41467-017-00674-6) 28993609 PMC5634439

[9] Graves DT, Milovanova TN. 2019 Mucosal immunity and the FOXO1 transcription factor. Front. Immunol. **10**, 2530. (10.3389/fimmu.2019.02530) 31849924 PMC6896163

[10] Delpoux A et al. 2018 Continuous activity of Foxo1 is required to prevent anergy and maintain the memory state of CD8^+^ T cells. J. Exp. Med. **215**, 575-594. (10.1084/jem.20170697) 29282254 PMC5789410

[11] Tzelepis F, Joseph J, Haddad EK, MacLean S, Dudani R, Agenes F, Peng SL, Sekaly R-P, Sad S. 2013 Intrinsic role of FoxO3a in the development of CD8+ T cell memory. J. Immunol. **190**, 1066-1075. (10.4049/jimmunol.1200639) 23277488 PMC3815477

[12] Chen Y, Liu Q, Guo D. 2020 Emerging coronaviruses: genome structure, replication, and pathogenesis. J. Med. Virol. **92**, 418-423. (10.1002/jmv.25681) 31967327 PMC7167049

[13] Wan Y, Shang J, Graham R, Baric RS, Li F. 2020 Receptor recognition by the novel coronavirus from Wuhan: an analysis based on decade-long structural studies of SARS coronavirus. J. Virol. **94**, e00127-20. 31996437 10.1128/JVI.00127-20 PMC7081895

[14] Imai Y et al. 2005 Angiotensin-converting enzyme 2 protects from severe acute lung failure. Nature **436**, 112-116. (10.1038/nature03712) 16001071 PMC7094998

[15] Kuba K et al. 2005 A crucial role of angiotensin converting enzyme 2 (ACE2) in SARS coronavirus-induced lung injury. Nat. Med. **11**, 875-879. (10.1038/nm1267) 16007097 PMC7095783

[16] Zhang X et al. 2020 Viral and host factors related to the clinical outcome of COVID-19. Nature **583**, 437-440. (10.1038/s41586-020-2355-0) 32434211

[17] Guan W et al. 2020 Clinical characteristics of coronavirus disease 2019 in China. N. Engl. J. Med. **382**, 1708-1720. (10.1056/NEJMoa2002032) 32109013 PMC7092819

[18] Wang C et al. 2020 Aveolar macrophage activation and cytokine storm in the pathogenesis of severe COVID-19. See www.immunology.ox.ac.uk/covid-19/covid-19-immunology-literature-reviews/alveolar-macrophage-activation-and-cytokine-storm-in-the-pathogenesis-of-severe-covid-19.

[19] Tian S, Hu W, Niu L, Liu H, Xu H, Xiao S-Y. 2020 Pulmonary pathology of early-phase 2019 novel coronavirus (COVID-19) pneumonia in two patients with lung cancer. J. Thorac. Oncol. **15**, 700-704. (10.1016/j.jtho.2020.02.010) 32114094 PMC7128866

[20] Wong RSM et al. 2003 Haematological manifestations in patients with severe acute respiratory syndrome: retrospective analysis. BMJ **326**, 1358-1362. (10.1136/bmj.326.7403.1358) 12816821 PMC162124

[21] Costela-Ruiz VJ, Illescas-Montes R, Puerta-Puerta JM, Ruiz C, Melguizo-Rodríguez L. 2020 SARS-CoV-2 infection: the role of cytokines in COVID-19 disease. Cytokine Growth Factor Rev. **54**, 62-75. 32513566 10.1016/j.cytogfr.2020.06.001 PMC7265853

[22] Yao Z, Zheng Z, Wu K, Junhua Z. 2020 Immune environment modulation in pneumonia patients caused by coronavirus: SARS-CoV, MERS-CoV and SARS-CoV-2. Aging *(*Albany NY) **12**, 7639. (10.18632/aging.103101) 32364527 PMC7244084

[23] Huang C et al. 2020 Clinical features of patients infected with 2019 novel coronavirus in Wuhan, China. Lancet **395**, 497-506. (10.1016/S0140-6736(20)30183-5) 31986264 PMC7159299

[24] Biondillo DE, Konicek SA, Iwamoto GK. 1994 Interferon-γ regulation of interleukin 6 in monocytic cells. Am. J. Physiol. Cell. Mol. Physiol. **267**, L564-L568. (10.1152/ajplung.1994.267.5.L564) 7526705

[25] Addeo A, Friedlaender A. 2020 Cancer and COVID-19: unmasking their ties. Cancer Treat. Rev. **88**, 102041. (10.1016/j.ctrv.2020.102041) 32516704 PMC7831797

[26] Schneider WM, Chevillotte MD, Rice CM. 2014 Interferon-stimulated genes: a complex web of host defenses. Annu. Rev. Immunol. **32**, 513-545. (10.1146/annurev-immunol-032713-120231) 24555472 PMC4313732

[27] Lam E-F, Francis RE, Petkovic M. 2006 FOXO transcription factors: key regulators of cell fate. Biochem. Soc. Trans. **34**, 722-726. 17052182 10.1042/BST0340722

[28] Brown AK, Webb AE. 2018 Regulation of FOXO factors in mammalian cells. Curr. Top. Dev. Biol. **127**, 165-192. (10.1016/bs.ctdb.2017.10.006) 29433737 PMC6383790

[29] Carracedo A, Pandolfi PP. 2008 The PTEN–PI3 K pathway: of feedbacks and cross-talks. Oncogene **27**, 5527-5541. (10.1038/onc.2008.247) 18794886

[30] Hornsveld M, Dansen TB, Derksen PW, Burgering BMT. 2018 Re-evaluating the role of FOXOs in cancer. In Seminars in cancer biology, pp. 90-100. Amsterdam, The Netherlands: Elsevier. 10.1016/j.semcancer.2017.11.017 29175105

[31] Martins R, Lithgow GJ, Link W. 2016 Long live FOXO: unraveling the role of FOXO proteins in aging and longevity. Aging Cell **15**, 196-207. (10.1111/acel.12427) 26643314 PMC4783344

[32] Wu C et al. 2020 Risk factors associated with acute respiratory distress syndrome and death in patients with coronavirus disease 2019 pneumonia in Wuhan, China. JAMA Intern. Med. **180**, 934-943. 32167524 10.1001/jamainternmed.2020.0994 PMC7070509

[33] Palumbo F, Seeger W, Morty RE. 2017 The role of FoxO transcription factors in normal and aberrant late lung development. Am. J. Resp. Crit. Care Med. **195**, A6394.

[34] Savai R et al. 2014 Pro-proliferative and inflammatory signaling converge on FoxO1 transcription factor in pulmonary hypertension. Nat. Med. **20**, 1289-1300. (10.1038/nm.3695) 25344740

[35] Xin Z, Ma Z, Hu W, Jiang S, Yang Z, Li T, Chen F, Jia G, Yang Y. 2018 FOXO1/3: potential suppressors of fibrosis. Ageing Res. Rev. **41**, 42-52. (10.1016/j.arr.2017.11.002) 29138094

[36] Malik S, Awasthi A. 2018 Transcriptional control of Th9 cells: role of foxo1 in interleukin-9 induction. Front. Immunol. **9**, 995. (10.3389/fimmu.2018.00995) 29867972 PMC5954031

[37] Ganesan S, Unger BL, Comstock AT, Angel KA, Mancuso P, Martinez FJ, Sajjan US. 2013 Aberrantly activated EGFR contributes to enhanced IL-8 expression in COPD airways epithelial cells via regulation of nuclear FoxO3A. Thorax **68**, 131-141. (10.1136/thoraxjnl-2012-201719) 23099361 PMC6712980

[38] Hwang J, Rajendrasozhan S, Yao H, Chung S, Sundar IK, Huyck HL, Pryhuber GS, Kinnula VL, Rahman I. 2011 FOXO3 deficiency leads to increased susceptibility to cigarette smoke-induced inflammation, airspace enlargement, and chronic obstructive pulmonary disease. J. Immunol. **187**, 987-998. (10.4049/jimmunol.1001861) 21690325 PMC3131437

[39] Maiese K, Chong ZZ, Shang YC. 2008 OutFOXOing disease and disability: the therapeutic potential of targeting FoxO proteins. Trends Mol. Med. **14**, 219-227. (10.1016/j.molmed.2008.03.002) 18403263 PMC2572150

[40] Kops GJPL et al. 2002 Forkhead transcription factor FOXO3a protects quiescent cells from oxidative stress. Nature **419**, 316-321. (10.1038/nature01036) 12239572

[41] Chiribau CB, Cheng L, Cucoranu IC, Yu Y-S, Clempus RE, Sorescu D. 2008 FOXO3A regulates peroxiredoxin III expression in human cardiac fibroblasts. J. Biol. Chem. **283**, 8211-8217. (10.1074/jbc.M710610200) 18195003 PMC2276380

[42] Ferber EC, Peck B, Delpuech O, Bell GP, East P, Schulze A. 2012 FOXO3a regulates reactive oxygen metabolism by inhibiting mitochondrial gene expression. Cell Death Differ. **19**, 968-979. (10.1038/cdd.2011.179) 22139133 PMC3354049

[43] Chattoraj SS, Ganesan S, Faris A, Comstock A, Lee W-M, Sajjan US. 2011 *Pseudomonas aeruginosa* suppresses interferon response to rhinovirus infection in cystic fibrosis but not in normal bronchial epithelial cells. Infect. Immun. **79**, 4131-4145. (10.1128/IAI.05120-11) 21825067 PMC3187241

[44] Lin L, Hron JD, Peng SL. 2004 Regulation of NF-κB, Th activation, and autoinflammation by the forkhead transcription factor Foxo3a. Immunity **21**, 203-213. (10.1016/j.immuni.2004.06.016) 15308101

[45] Wu H, Zhang R, Fan X, Lian Z, Hu Y. 2019 FoxOs could play an important role during influenza A viruses infection via microarray analysis based on GEO database. Infect. Genet. Evol. **75**, 104009. (10.1016/j.meegid.2019.104009) 31437558

[46] Lei C-Q, Zhang Y, Xia T, Jiang L-Q, Zhong B, Shu H-B. 2013 FoxO1 negatively regulates cellular antiviral response by promoting degradation of IRF3. J. Biol. Chem. **288**, 12 596-12 604. (10.1074/jbc.M112.444794) PMC3642307 23532851

[47] Liu X, Cai X, Zhang D, Xu C, Xiao W. 2016 Zebrafish foxo3b negatively regulates antiviral response through suppressing the transactivity of irf3 and irf7. J. Immunol. **197**, 4736-4749. (10.4049/jimmunol.1601187) 27815423

[48] Kim TH, Zhou H. 2018 Overexpression of chicken IRF7 increased viral replication and programmed cell death to the avian influenza virus infection through TGF-Beta/FoxO signaling axis in DF-1. Front. Genet. **9**, 415. (10.3389/fgene.2018.00415) 30356848 PMC6190866

[49] Litvak V et al. 2012 A FOXO3–IRF7 gene regulatory circuit limits inflammatory sequelae of antiviral responses. Nature **490**, 421-425. (10.1038/nature11428) 22982991 PMC3556990

[50] Gimenes-Junior J, Owuar N, Vari HR, Li W, Xander N, Kotnala S, Sajjan US. 2019 FOXO3a regulates rhinovirus-induced innate immune responses in airway epithelial cells. Sci. Rep. **9**, 18180. (10.1038/s41598-019-54567-3) 31796819 PMC6890790

[51] Mehta PK, Griendling KK. 2007 Angiotensin II cell signaling: physiological and pathological effects in the cardiovascular system. Am. J. Physiol. Physiol. **292**, C82-C97. (10.1152/ajpcell.00287.2006) 16870827

[52] Magrone T, Magrone M, Jirillo E. 2020 Focus on receptors for coronaviruses with special reference to angiotensin-converting enzyme 2 as a potential drug target-a perspective. Endocrine, Metab. Immune Disord. Targets (Formerly Curr. Drug Targets-Immune, Endocr. Metab. Disord) **20**, 807-811. (10.2174/1871530320666200427112902) 32338224

[53] Monteil V et al. 2020 Inhibition of SARS-CoV-2 infections in engineered human tissues using clinical-grade soluble human ACE2. Cell. **181**, 905-913. 32333836 10.1016/j.cell.2020.04.004 PMC7181998

[54] Pedersen KB, Chodavarapu H, Lazartigues E. 2017 Forkhead box transcription factors of the FOXA class are required for basal transcription of angiotensin-converting enzyme 2. J. Endocr. Soc. **1**, 370-384. (10.1210/js.2016-1071) 29082356 PMC5656262

[55] Perkins GD, Gao F, Thickett DR. 2008 In vivo and in vitro effects of salbutamol on alveolar epithelial repair in acute lung injury. Thorax **63**, 215-220. (10.1136/thx.2007.080382) 17951278

[56] Geiser T, Atabai K, Jarreau P-H, Ware LB, Pugin J, Matthay MA. 2001 Pulmonary edema fluid from patients with acute lung injury augments in vitro alveolar epithelial repair by an IL-1 β-dependent mechanism. Am. J. Respir. Crit. Care Med. **163**, 1384-1388. (10.1164/ajrccm.163.6.2006131) 11371405

[57] Clarke NE, Belyaev ND, Lambert DW, Turner AJ. 2014 Epigenetic regulation of angiotensin-converting enzyme 2 (ACE2) by SIRT1 under conditions of cell energy stress. Clin. Sci. **126**, 507-516. (10.1042/CS20130291) 24147777

[58] Hori YS, Kuno A, Hosoda R, Horio Y. 2013 Regulation of FOXOs and p53 by SIRT1 modulators under oxidative stress. PLoS ONE **8**, e73875. (10.1371/journal.pone.0073875) 24040102 PMC3770600

[59] Nakagawa K, Lokugamage KG, Makino S. 2016 Viral and cellular mRNA translation in coronavirus-infected cells. Adv. Virus Res. **96**, 165-192. 27712623 10.1016/bs.aivir.2016.08.001 PMC5388242

[60] Krähling V, Stein DA, Spiegel M, Weber F, Mühlberger E. 2009 Severe acute respiratory syndrome coronavirus triggers apoptosis via protein kinase R but is resistant to its antiviral activity. J. Virol. **83**, 2298-2309. (10.1128/JVI.01245-08) 19109397 PMC2643707

[61] Pinto BGG, Oliveira AER, Singh Y, Jimenez L, Gonçalves ANA, Ogava RLT, Creighton R, Peron JPS, Nakaya HI. 2020 ACE2 expression is increased in the lungs of patients with comorbidities associated with severe COVID-19. J. Infect. Dis. **222**, 556-563. 32526012 10.1093/infdis/jiaa332 PMC7377288

[62] Ghosh HS, Reizis B, Robbins PD. 2011 SIRT1 associates with eIF2-α and regulates the cellular stress response. Sci. Rep. **1**, 150. (10.1038/srep00150) 22355666 PMC3252071

[63] Smith K. 2020 Novel coronavirus: hypothesis of treatment with SIRT1 inhibitors. OSF Preprints. (10.31219/osf.io/h62tz

[64] Kobayashi Y, Furukawa-Hibi Y, Chen C, Horio Y, Isobe K, Ikeda K, Motoyama N. 2005 SIRT1 is critical regulator of FOXO-mediated transcription in response to oxidative stress. Int. J. Mol. Med. **16**, 237-243. (10.3892/ijmm.16.2.237 16012755

[65] Chan C-P, Siu K-L, Chin K-T, Yuen K-Y, Zheng B, Jin D-Y. 2006 Modulation of the unfolded protein response by the severe acute respiratory syndrome coronavirus spike protein. J. Virol. **80**, 9279-9287. (10.1128/JVI.00659-06) 16940539 PMC1563899

[66] Zhang W, Hietakangas V, Wee S, Lim SC, Gunaratne J, Cohen SM. 2013 ER stress potentiates insulin resistance through PERK-mediated FOXO phosphorylation. Genes Dev. **27**, 441-449. (10.1101/gad.201731.112) 23431056 PMC3589560

[67] You S, Li H, Hu Z, Zhang W. 2018 eIF 2*α* kinases PERK and GCN 2 act on FOXO to potentiate FOXO activity. Genes Cells **23**, 786-793. (10.1111/gtc.12625) 30043468

[68] Furukawa M, Xiong Y. 2005 BTB protein Keap1 targets antioxidant transcription factor Nrf2 for ubiquitination by the Cullin 3-Roc1 ligase. Mol. Cell. Biol. **25**, 162-171. (10.1128/MCB.25.1.162-171.2005) 15601839 PMC538799

[69] Itoh K et al. 1997 An Nrf2/small Maf heterodimer mediates the induction of phase II detoxifying enzyme genes through antioxidant response elements. Biochem. Biophys. Res. Commun. **236**, 313-322. (10.1006/bbrc.1997.6943) 9240432

[70] Deshmukh P, Unni S, Krishnappa G, Padmanabhan B. 2017 The Keap1–Nrf2 pathway: promising therapeutic target to counteract ROS-mediated damage in cancers and neurodegenerative diseases. Biophys. Rev. **9**, 41-56. (10.1007/s12551-016-0244-4) 28510041 PMC5425799

[71] Guan L, Zhang L, Gong Z, Hou X, Xu Y, Feng X, Wang H, You H. 2016 FoxO3 inactivation promotes human cholangiocarcinoma tumorigenesis and chemoresistance through Keap1-Nrf2 signaling. Hepatology **63**, 1914-1927. (10.1002/hep.28496) 26857210

[72] Olagnier DP et al. 2020 Identification of SARS-CoV2-mediated suppression of NRF2 signaling reveals a potent antiviral and anti-inflammatory activity of 4-octyl-itaconate and dimethyl fumarate. Nat. Commun. **11**, 4938. (10.1038/s41467-020-18764-3) 33009401 PMC7532469

[73] Kang J, Jeong MG, Oh S, Jang EJ, Kim HK, Hwang ES. 2014 A FoxO1-dependent, but NRF2-independent induction of heme oxygenase-1 during muscle atrophy. FEBS Lett. **588**, 79-85. (10.1016/j.febslet.2013.11.009) 24269680

[74] Espinoza JA, González PA, Kalergis AM. 2017 Modulation of antiviral immunity by heme oxygenase-1. Am. J. Pathol. **187**, 487-493. (10.1016/j.ajpath.2016.11.011) 28082120

[75] Koliaraki V, Kollias G. 2011 A new role for myeloid HO-1 in the innate to adaptive crosstalk and immune homeostasis. Adv. Exp. Med. Biol. **780**, 101-111. 21842368 10.1007/978-1-4419-5632-3_9

[76] Wagener FA, Pickkers P, Peterson SJ, Immenschuh S, Abraham NG. 2020 Targeting the heme-heme oxygenase system to prevent severe complications following COVID-19 infections. Antioxidants **9**, 540. (10.3390/antiox9060540) 32575554 PMC7346191

[77] Hooper PL. 2020 COVID-19 and heme oxygenase: novel insight into the disease and potential therapies. Cell Stress Chaperones **25**, 707-710. (10.1007/s12192-020-01126-9) 32500379 PMC7271958

[78] DeDiego ML, Nieto-Torres JL, Regla-Nava JA, Jimenez-Guardeño JM, Fernandez-Delgado R, Fett C, Castaño-Rodriguez C, Perlman S, Enjuanes L. 2014 Inhibition of NF-κB-mediated inflammation in severe acute respiratory syndrome coronavirus-infected mice increases survival. J. Virol. **88**, 913-924. (10.1128/JVI.02576-13) 24198408 PMC3911641

[79] Dosch SF, Mahajan SD, Collins AR. 2009 SARS coronavirus spike protein-induced innate immune response occurs via activation of the NF-κB pathway in human monocyte macrophages in vitro. Virus Res. **142**, 19-27. (10.1016/j.virusres.2009.01.005) 19185596 PMC2699111

[80] Lee H-Y et al. 2008 FOXO3a turns the tumor necrosis factor receptor signaling towards apoptosis through reciprocal regulation of c-Jun N-terminal kinase and NF-κB. Arterioscler. Thromb. Vasc. Biol. **28**, 112-120. (10.1161/ATVBAHA.107.153304) 18032780

[81] Kauppinen A, Suuronen T, Ojala J, Kaarniranta K, Salminen A. 2013 Antagonistic crosstalk between NF-κB and SIRT1 in the regulation of inflammation and metabolic disorders. Cell Signal. **25**, 1939-1948. (10.1016/j.cellsig.2013.06.007) 23770291

[82] Lang J, Yang N, Deng J, Liu K, Yang P, Zhang G, Jiang C. 2011 Inhibition of SARS pseudovirus cell entry by lactoferrin binding to heparan sulfate proteoglycans. PLoS ONE **6**, e23710. (10.1371/journal.pone.0023710) 21887302 PMC3161750

[83] Chang R, Ng TB, Sun W-Z. 2020 Lactoferrin as potential preventative and adjunct treatment for COVID-19. Int. J. Antimicrob. Agents **56**, 106118. (10.1016/j.ijantimicag.2020.106118) 32738305 PMC7390755

[84] Wu X, Fan Z, Chen M, Chen Y, Rong D, Cui Z, Yuan Y, Zhuo L, Xu Y. 2019 Forkhead transcription factor FOXO 3a mediates interferon-*γ*-induced MHC II transcription in macrophages. Immunology **158**, 304-313. (10.1111/imm.13116) 31509237 PMC6856938

[85] Bruchez A et al. 2020 MHC class II transactivator CIITA induces cell resistance to Ebola virus and SARS-like coronaviruses. Science **370**, 241-247. (10.1126/science.abb3753) 32855215 PMC7665841

[86] Acharya D, Liu G, Gack MU. 2020 Dysregulation of type I interferon responses in COVID-19. Nat. Rev. Immunol. **20**, 397-398. 32457522 10.1038/s41577-020-0346-x PMC7249038

[87] Hensley LE, Fritz EA, Jahrling PB, Karp C, Huggins JW, Geisbert TW. 2004 Interferon-β 1a and SARS coronavirus replication. Emerg. Infect. Dis. **10**, 317. (10.3201/eid1002.030482) 15030704 PMC3322919

[88] Nile SH, Nile A, Qiu J, Li L, Jia X, Kai G. 2020 COVID-19: pathogenesis, cytokine storm and therapeutic potential of interferons. Cytokine Growth Factor Rev. **53**, 66-70. 32418715 10.1016/j.cytogfr.2020.05.002 PMC7204669

[89] Perumal NB, Kaplan MH. 2011 Regulating Il9 transcription in T helper cells. Trends Immunol. **32**, 146-150. (10.1016/j.it.2011.01.006) 21371941 PMC3070825

[90] Peng SL. 2010 Forkhead transcription factors in chronic inflammation. Int. J. Biochem. Cell Biol. **42**, 482-485. (10.1016/j.biocel.2009.10.013) 19850149 PMC2835811

[91] Delgado-Roche L, Mesta F. 2020 Oxidative stress as key player in severe acute respiratory syndrome coronavirus (SARS-CoV) infection. Arch. Med. Res. **51**, 384-387. 32402576 10.1016/j.arcmed.2020.04.019 PMC7190501

[92] Hu R, Xu H, Jiang H, Zhang Y, Sun Y. 2013 The role of TLR4 in the pathogenesis of indirect acute lung injury. Front. Biosci. (Landmark Ed). **18**, 1244-1255. (10.2741/4176) 23747880

[93] Marfè G, Tafani M, Fiorito F, Pagnini U, Iovane G, De Martino L. 2011 Involvement of FOXO transcription factors, TRAIL-FasL/Fas, and sirtuin proteins family in canine coronavirus type II-induced apoptosis. PLoS ONE **6**, e27313. (10.1371/journal.pone.0027313) 22087287 PMC3210785

[94] Sridhar S et al. 2013 Cellular immune correlates of protection against symptomatic pandemic influenza. Nat. Med. **19**, 1305. (10.1038/nm.3350) 24056771

[95] Choe PG et al. 2017 MERS-CoV antibody responses 1 year after symptom onset, South Korea, 2015. Emerg. Infect. Dis. **23**, 1079. (10.3201/eid2307.170310) PMC5512479 28585916

[96] Okba NMA et al. 2019 Sensitive and specific detection of low-level antibody responses in mild middle east respiratory syndrome coronavirus infections. Emerg. Infect. Dis. **25**, 1868. (10.3201/eid2510.190051) 31423970 PMC6759241

[97] Zhao J et al. 2017 Recovery from the Middle East respiratory syndrome is associated with antibody and T cell responses. Sci. Immunol. **2**, eaan5393. (10.1126/sciimmunol.aan5393) 28778905 PMC5576145

[98] Channappanavar R, Zhao J, Perlman S. 2014T cell-mediated immune response to respiratory coronaviruses. Immunol. Res. **59**, 118-128. (10.1007/s12026-014-8534-z) 24845462 PMC4125530

[99] Dan JM et al. 2021 Immunological memory to SARS-CoV-2 assessed for up to 8 months after infection. Science **371**, eabf4063. 33408181 10.1126/science.abf4063 PMC7919858

[100] García LF. 2020 Immune response, inflammation, and the clinical spectrum of COVID-19. Front. Immunol. **11**, 1441. (10.3389/fimmu.2020.01441) 32612615 PMC7308593

[101] Newton RH et al. 2018 Maintenance of CD4T cell fitness through regulation of Foxo1. Nat. Immunol. **19**, 838-848. (10.1038/s41590-018-0157-4) 29988091 PMC6289177

[102] Aksoylar HI, Lampe K, Barnes MJ, Plas DR, Hoebe K. 2012 Loss of immunological tolerance in Gimap5-deficient mice is associated with loss of Foxo in CD4^+^ T cells. J. Immunol. **188**, 146-154. (10.4049/jimmunol.1101206) 22106000 PMC3258489

[103] Ouyang W et al. 2012 Novel Foxo1-dependent transcriptional programs control T reg cell function. Nature **491**, 554-559. (10.1038/nature11581) 23135404 PMC3771531

[104] Utzschneider DT, Delpoux A, Wieland D, Huang X, Lai C-Y, Hofmann M, Thimme R, Hedrick SM. 2018 Active maintenance of T cell memory in acute and chronic viral infection depends on continuous expression of FOXO1. Cell Rep. **22**, 3454-3467. (10.1016/j.celrep.2018.03.020) 29590615 PMC5942184

[105] Michelini RH, Doedens AL, Goldrath AW, Hedrick SM. 2013 Differentiation of CD8 memory T cells depends on Foxo1. J. Exp. Med. **210**, 1189-1200. (10.1084/jem.20130392) 23712431 PMC3674697

[106] Kim MV, Ouyang W, Liao W, Zhang MQ, Li MO. 2013 The transcription factor Foxo1 controls central-memory CD8^+^ T cell responses to infection. Immunity **39**, 286-297. (10.1016/j.immuni.2013.07.013) 23932570 PMC3809840

[107] Inoue T, Shinnakasu R, Ise W, Kawai C, Egawa T, Kurosaki T. 2017 The transcription factor Foxo1 controls germinal center B cell proliferation in response to T cell help. J. Exp. Med. **214**, 1181-1198. (10.1084/jem.20161263) 28351982 PMC5379976

[108] Cabrera-Ortega AA, Feinberg D, Liang Y, Rossa Jr C, Graves DT 2017 The role of forkhead box 1 (FOXO1) in the immune system: dendritic cells, T cells, B cells, and hematopoietic stem cells. Crit. Rev. Immunol. **37**, 1-13. 29431075 10.1615/CritRevImmunol.2017019636 PMC6085137

[109] Sullivan JA, Kim EH, Plisch EH, Peng SL, Suresh M. 2012 FOXO3 regulates CD8 T cell memory by T cell-intrinsic mechanisms. PLoS Pathog. **8**, e1002533. (10.1371/journal.ppat.1002533) 22359505 PMC3280979

[110] Dejean AS, Beisner DR, Ch'En IL, Kerdiles YM, Babour A, Arden KC, Castrillon DH, DePinho RA, Hedrick SM. 2009 Transcription factor Foxo3 controls the magnitude of T cell immune responses by modulating the function of dendritic cells. Nat. Immunol. **10**, 504-513. (10.1038/ni.1729) 19363483 PMC2712214

[111] Mahase E. 2020 Covid-19: demand for dexamethasone surges as RECOVERY trial publishes preprint. Br. Med. J. **369**, m2512. (10.1136/bmj.m2512) 32576548

[112] Theoharides TC, Conti P. 2020 Dexamethasone for COVID-19? Not so fast. J. Biol. Regul. Homeost. Agents **34**, 1241-1243. (10.23812/20-EDITORIAL_1-5) 32551464

[113] Antwi-Amoabeng D, Kanji Z, Ford B, Beutler BD, Riddle MS, Siddiqui F. 2020 Clinical outcomes in COVID-19 patients treated with tocilizumab: an individual patient data systematic review. J. Med. Virol. **92**, 2516-2522. 32436994 10.1002/jmv.26038 PMC7280615

[114] González-García A, García-Sánchez I, Lopes V, Moreno-Arrones OM, Tortosa-Cabañas M, Elías-Sáenz I, Hernández-Rodríguez J. 2020 Successful treatment of severe COVID-19 with subcutaneous anakinra as a sole treatment. Rheumatology **59**, 2171-2173. (10.1093/rheumatology/keaa318) 32568376 PMC7337866

[115] De Biasi S et al. 2020 Marked T cell activation, senescence, exhaustion and skewing towards TH17 in patients with COVID-19 pneumonia. Nat. Commun. **11**, 1-17. (10.1038/s41467-020-17292-4) 32632085 PMC7338513

[116] Cautain B et al. 2016 Discovery of a novel, isothiazolonaphthoquinone-based small molecule activator of FOXO nuclear-cytoplasmic shuttling. PLoS ONE **11**, e0167491. (10.1371/journal.pone.0167491) 27936162 PMC5147912

[117] Yang J-Y, Hung M-C. 2009 A new fork for clinical application: targeting forkhead transcription factors in cancer. Clin. Cancer Res. **15**, 752-757. (10.1158/1078-0432.CCR-08-0124) 19188143 PMC2676228

[118] Lu H, Liu P, Pan Y, Huang H. 2011 Inhibition of cyclin-dependent kinase phosphorylation of FOXO1 and prostate cancer cell growth by a peptide derived from FOXO1. Neoplasia **13**, 854-863. (10.1593/neo.11594) 21969818 PMC3182277

[119] Zhang H, Dong Y, Zhao H, Brooks JD, Hawthorn L, Nowak N, Marshall JR, Gao AC, Ip C. 2005 Microarray data mining for potential selenium targets in chemoprevention of prostate cancer. Cancer Genomics Proteomics **2**, 97-113. 18548127 PMC2424238

[120] Schroeder FC, Kau TR, Silver PA, Clardy J. 2005 The psammaplysenes, specific inhibitors of FOXO1a nuclear export. J. Nat. Prod. **68**, 574-576. (10.1021/np049624z) 15844952

[121] Corno C et al. 2018 FoxO-1 contributes to the efficacy of the combination of the XPO1 inhibitor selinexor and cisplatin in ovarian carcinoma preclinical models. Biochem. Pharmacol. **147**, 93-103. (10.1016/j.bcp.2017.11.009) 29155058

[122] Wymann MP, Bulgarelli-Leva G, Zvelebil MJ, Pirola L, Vanhaesebroeck B, Waterfield MD, Panayotou G. 1996 Wortmannin inactivates phosphoinositide 3-kinase by covalent modification of Lys-802, a residue involved in the phosphate transfer reaction. Mol. Cell. Biol. **16**, 1722-1733. (10.1128/MCB.16.4.1722) 8657148 PMC231159

[123] Vlahos CJ, Matter WF, Hui KY, Brown RF. 1994 A specific inhibitor of phosphatidylinositol 3-kinase, 2-(4-morpholinyl)-8-phenyl-4H-1-benzopyran-4-one (LY294002). J. Biol. Chem. **269**, 5241-5248. (10.1016/S0021-9258(17)37680-9) 8106507

[124] Ihle NT et al. 2004 Molecular pharmacology and antitumor activity of PX-866, a novel inhibitor of phosphoinositide-3-kinase signaling. Mol. Cancer Ther. **3**, 763-772. 15252137

[125] Forni G, Mantovani A. 2021 COVID-19 vaccines: where we stand and challenges ahead. Cell Death Differ. **28**, 626-639. (10.1038/s41418-020-00720-9) 33479399 PMC7818063

[126] Kim S et al. 2020 The progression of sars coronavirus 2 (sars-cov2): mutation in the receptor binding domain of spike gene. Immune Netw. **20**, E41. (10.4110/in.2020.20.e41) 33163249 PMC7609167

[127] Greaney AJ et al. 2021 Complete mapping of mutations to the SARS-CoV-2 spike receptor-binding domain that escape antibody recognition. Cell Host Microbe **29**, 44-57. (10.1016/j.chom.2020.11.007) 33259788 PMC7676316

[128] Tu Y-F et al. 2020 A review of SARS-CoV-2 and the ongoing clinical trials. Int. J. Mol. Sci. **21**, 2657. (10.3390/ijms21072657) 32290293 PMC7177898

[129] Carr AC, Rowe S. 2020 The emerging role of vitamin C in the prevention and treatment of COVID-19. Nutrients **12**, 3286. (10.3390/nu12113286) 33121019 PMC7693980

